# Recombinant Human Parathyroid Hormone Related Protein 1-34 and 1-84 and Their Roles in Osteoporosis Treatment

**DOI:** 10.1371/journal.pone.0088237

**Published:** 2014-02-06

**Authors:** Hua Wang, Jingning Liu, Ying Yin, Jun Wu, Zilu Wang, Dengshun Miao, Wen Sun

**Affiliations:** 1 The Research Center for Bone and Stem Cells, Department of Anatomy, Histology and Embryology, Nanjing Medical University, Nanjing, P.R. of China; 2 Institute of Dental Research, Stomatological College, Nanjing Medical University, Nanjing, P.R. of China; 3 Department of Geriatrics, The Second Affiliated Hospital of Nanjing Medical University, Nanjing, P.R. of China; University of Ulm, Germany

## Abstract

Osteoporosis is a common disorder characterized by compromised bone strength that predisposes patients to increased fracture risk. Parathyroid hormone related protein (PTHrP) is one of the candidates for clinical osteoporosis treatment. In this study, GST Gene Fusion System was used to express recombinant human PTHrP (hPTHrP) 1-34 and 1-84. To determine whether the recombinant hPTHrP1-34 and 1-84 can enhance renal calcium reabsorption and promote bone formation, we examined effects of recombinant hPTHrP1-34 and 1-84 on osteogenic lineage commitment in a primary bone marrow cell culture system and on osteoporosis treatment. Results revealed that both of recombinant hPTHrP1-34 and 1-84 increased colony formation and osteogenic cell differentiation and mineralization in vitro; however, the effect of recombinant hPTHrP1-84 is a little stronger than that of hPTHrP1-34. Next, ovariectomy was used to construct osteoporosis animal model (OVX) to test activities of these two recombinants in vivo. HPTHrP1-84 administration elevated serum calcium by up-regulating the expression of renal calcium transporters, which resulted in stimulation of osteoblastic bone formation. These factors contributed to augmented bone mass in hPTHrP1-84 treated OVX mice but did not affect bone resorption. There was no obvious bone mass alteration in hPTHrP1-34 treated OVX mice, which may be, at least partly, associated with shorter half-life of hPTHrP1-34 compared to hPTHrP1-84 in vivo. This study implies that recombinant hPTHrP1-84 is more effective than hPTHrP1-34 to enhance renal calcium reabsorption and to stimulate bone formation in vivo.

## Introduction

Osteoporosis is a common disorder characterized by compromised bone strength that predisposes patients to increased fracture risk. It arises, in part, as a consequence of bone loss secondary to an imbalance between the processes of bone resorption and formation during the normal bone remodeling cycle [1]. Clinical practice revealed that postmenopausal osteoporosis and age-related osteoporosis are the most common primary forms of bone loss.

A variety of drugs are now available for osteoporosis treatment, including antiresorptive and anabolic agents. Antiresorptive drugs treating osteoporosis, act by inhibiting osteoclast activity and consequently slowing the rate of bone remodeling, rather than by rebuilding bone. Antiresorptive drugs including estrogens, the selective estrogen receptor modulator, raloxifene, bisphosphonates and calcitonin [2]. Besides antiresorptive agents, the usage of anabolic agents is an important new advancement in osteoporosis treatment. Anabolic agents reduce fracture incidence by directly stimulating bone formation in addition to increasing bone mass. The only anabolic agent currently being approved in the United States for osteoporosis treatment, parathyroid hormone 1-34 (PTH1-34, teriparatide), has emerged as a major therapeutic approach to treat osteoporosis. PTH normally regulates serum calcium levels by binding and activating the type 1 PTH receptor (PTHR1) in bone and kidney [3]. As long as the dose and pattern of administration are carefully selected, exogenous PTH can stimulate bone formation in animals and in humans [2], [4]. However, PTH has its side effect, which stimulates cortical bone loss [5]. Besides PTH, there are also some other potential anabolic agents for osteoporosis treatment, including parathyroid hormone related protein (PTHrP), growth hormone, insulin-like growth factor-1 (IGF-1), and so on [6]–[7]. Among these drugs, PTHrP has gained more and more attention in recent years [8].

PTHrP, a factor isolated from tumors associated with the paraneoplastic syndrome of humoral hypercalcemia of malignancy, shares homology with PTH1-34 [9]–[10]. Mice heterozygous for the *PTHrP*-null allele (*PTHrP^+/−^* mice) exhibit a form of skeletal haploinsufficiency characterized by decreased bone volume and bony structural alterations consistent with premature, advanced osteoporosis, which means PTHrP is necessary for bone remodeling [11]. Our previous studies have demonstrated that PTHrP is a key factor in bone formation [12]–[13]. PTHrP is a poly-hormone which can be translated and processed into many smaller bioactive forms, including an N-terminal peptide, midregion, nuclear localization sequence (NLS) and C-terminal region. The N-terminal PTHrP1-34 is structurally and functionally similar to PTH. The mid-region PTHrP35-84 or 86 exhibits a major role in placental calcium transport. The amino acids 88-107 region of NLS, targets PTHrP to the nucleus, where it regulates cell differentiation and apoptosis through an intracrine pathway. The C-terminal PTHrP108-141 acts as osteoclast activity inhibition [14]–[17].

Consistent with this concept, recombinant PTHrP1-84 treatment caused an increase in placental calcium transfer, whereas synthetic PTHrP1-34 had no such effect [18]. It supports the concept that the mid-molecule region of PTHrP is functionally important in placental calcium transport. Our previous studies also demonstrated that PTHrP1-86 can enhanced renal calcium reabsorption by promoting calcium transporters expression in kidney [19]. However, it is unclear whether there are some differences between PTHrP1-34 and 1-84 on renal calcium reabsorption and on bone formation. To answer this question, GST Gene Fusion System was used to express recombinant human PTHrP1-34 (hPTHrP1-34) and 1-84 (hPTHrP1-84). We tested the hypothesis that the recombinant hPTHrP1-84 is more effective than hPTHrP1-34 to enhance renal calcium reabsorption and to stimulate bone formation. We used the bilateral ovariectomized mice as osteoporosis animal model to test this hypothesis. We also explored the mechanism of the effect of recombinant N-terminal domain of PTHrP (hPTHrP1-34 and 1-84) on bone and kidney in this setting.

## Materials and Methods

The use of animals in this study was approved by the Institutional Animal Care and Use Committee of Nanjing Medical University (Approval ID 2008-00318).

### Cloning and expression of *hPTHrP1-34* and *1-84* in *pGEX-2TK*

The *hPTHrP1-34* and *1-84* fragments were amplified by RT-PCR from human bony complementary DNA using P1 (5′- TTTCCGGGATCCATGGCTGTGTCTGAACATCAGCT)/P2 (5′- TTTCCGGAATTCTCAAGCTGTGTGGATTTCTGCG) and P3 (5′- TTTCCGGGATCCATGGCTGTGTCTGAACATCAGCT)/P4 (5′- TTTCCGGAATTCTCACTTGAGCGGCTGCTCTTTG) primers, and the resulting product was digested with BamHI and EcoRI (sites underlined) and ligated into the GST fusion vector *pGEX-2TK* vector (Amersham). The recombinant DNA was used to transform competent E. coli BL21 (DE3) strains. Ampicillin resistance colonies were grown overnight in agar plate containing 50 µg/ml ampicillin, diluted ten times in the same medium, and grown to a concentration of 1 absorbance U/ml (at 600 nm). Expression of hPTHrP1-34 and 1-84 were induced with 1 mM isopropyl-beta-D-thiogalactopyranoside (IPTG) for 0h, 1h, 2h, 3h or 4h, and the bacterial suspension were harvested. The cells were pelleted, resuspended at 1/20 volume in cold 1×PBS, and sonicated on ice. Total protein from soluble fractions was dissolved in 2×SDS-PAGE buffer (2% SDS, 20% glycerol, 0.001% Bromphenol blue, and 0.125 M Tris-HCl pH 6.8) with subsequent boiling and fractionated by SDS-PAGE.

### Purification of hPTHrP1-34 and 1-84

Purification of soluble hPTHrP1-34 and 1-84 separately from 1 liter culture of E. coli BL21 were performed on Glutathione Sepharose 4B columns (Amersham) using the recommended procedure. The fusion protein was digested 4h on the column with 20 U thrombin (Amersham) and eluted with 1 ml 1×PBS. Binding and elution steps were carried out in the cold to decrease proteolytic degradation of hPTHrP1-34 and 1-84. Purified hPTHrP1-34 and 1-84 were quantitated by a kit (Bio-Rad, Mississauga, Ontario, Canada) and prepared for Western blotting, in vitro and in vivo experiments as described below.

### Primary bone marrow cell cultures

Tibiae and femurs of 200-g male Wistar rats were removed under aseptic conditions, and bone marrow cells (BMC) were flushed out with Dulbecco's modified Eagle's minimal essential medium containing 10% fetal calf serum, 50 µg/ml ascorbic acid, 10 mM β-glycerophosphate, and 10^−8^ M dexamethasone. Cells were dispersed by repeated pipetting, and a single-cell suspension was achieved by forcefully expelling the cells through a 22-gauge syringe needle. 10^6^ total bone marrow cells were cultured in 36-cm^2^ Petri dishes in 5 ml of the above-mentioned medium. All the dishes were assigned to three different groups. Each of the group treated with vehicle (Control), 10^−7^ M hPTHrP1-34 or 10^−7^ M hPTHrP1-84. The medium was changed every 4 days. The non-adherent cells containing hematopoietic elements were removed by pipetting gently when the medium was changed for the first time [20]. Only monocytic cells remained. Cultures were maintained for 12–18 days. At the end of the culture period cells were stained cytochemically or RNA was extracted from these cells for RT-PCR as described below.

### Cytochemical staining

The cells from 18-day primary BMC cultures were washed with PBS and fixed by the addition of cold ethanol. After fixation, the cultures were stained sequentially for alkaline phosphatase (ALP)-positive colonies, calcified colonies, collagen-positive colonies, and total colonies as described previously [21]. After each staining procedure, the culture dishes were photographed, destained, and then restained as required. The numbers of colonies were counted macroscopically by hand. Total fibroblastic colonies, *i.e.* colonies predominantly containing cells with a mesenchymal morphology, were considered to represent colony forming units fibroblastic (CFU-f). Colonies that also stained positive for ALP, calcium, and/or collagen were considered to be derived from CFU-f with the ability to express ALP, calcium, and/or synthesize collagen and were termed CFU-f _ALP_, CFU-f _Ca_, and CFU-f _Col_.

### In vivo experiments

Twenty four female C57BL/6J mice, 8 weeks age, were assigned to four different groups, with six mice per group. All mice were anesthetized with chloral hydrate (0.4 ml/100 g body weight) and subjected to surgery on day 0. Sham surgery was performed on the Sham group (Sham) by identifying the bilateral ovaries and ovariectomy (OVX) was performed on the other groups by removing the bilateral ovaries. After surgery, all mice were maintained in a virus- and parasite-free barrier facility and exposed to a 12-h/12-h light/dark cycle under standard conditions in the Medical Experimental Animal Center of Nanjing Medical University for 9 weeks. HPTHrP1–34 was subcutaneously administered at a dose of 10 nmol/kg (40 µg/kg) body weight everyday for the last 4 weeks. HPTHrP1–84 was subcutaneously administered at a dose of 10 nmol/kg (100 µg/kg) body weight everyday for the last 4 weeks. Each of the mice received vehicle (vehicle-treated sham-operated mice and vehicle-treated OVX mice), hPTHrP1-34 (hPTHrP1-34-treated OVX mice) or hPTHrP1-84 (hPTHrP1-84-treated OVX mice). At the end of experiments, all mice were sacrificed by exsanguination under chloral hydrate anesthesia and blood were collected for serum calcium, phosphorus and ALP analyses, bones and kidneys were then removed for the analyses described below.

### Serum PTHrP analyses

The OVX mice were injected with vehicle or 10 nmol/kg hPTHrP1-34 or 10 nmol/kg hPTHrP1-84 subcutaneously. Subsequently, blood samples were drawn at 15 and 30 minutes, 1, 2, 4 and 6 hours after the injections. Serum concentrations of hPTHrP1-34 or hPTHrP1-84 were measured by enzyme linked immunosorbent assay (ELISA) that used Phoenix Pharmaceuticals PTHrP(1-34)- EIA Kit (Phoenix Pharmaceuticals, USA). Procedures were performed according to the instructions of the kit.

### Serum calcium, phosphorus and ALP analyses

Serum calcium, phosphorus and ALP were determined at 4 weeks after the administration of drugs by autoanalyzer (Beckman Synchron 67; Beckman Instruments).

### Radiography

Thoracic vertebrae were removed and dissected free of soft tissue. Contact radiographs were taken using a Faxitron model 805 radiographic inspection system (Faxitron Contact, Faxitron, Germany) (22 kV voltage and 4 mins exposure time). X-Omat TL film (Eastman Kodak Co., Rochester, NY, USA) was used and processed routinely.

### Micro-computed tomography (micro-CT)

Thoracic vertebrae were fixed overnight in 70% ethanol and analyzed by micro-CT with a SkyScan 1072 scanner and associated analysis software (SkyScan, Antwerp, Belgium) as described [22]. Briefly, image acquisition was performed at 100 kV and 98 µA with a 0.9° rotation between frames. During scanning, the samples were enclosed in tightly fitting plastic wrap to prevent movement and dehydration. Thresholding was applied to the images to segment the bone from the background. Two-dimensional images were used to generate three-dimensional renderings using the 3D Creator software supplied with the instrument. The resolution of the micro-CT images is 18.2 µm.

### Histology

Lumbar vertebrae or tibiae were removed and fixed in PLP fixative (2% paraformaldehyde containing 0.075 M lysine and 0.01 M sodium periodate solution) overnight at 4°C and processed histologically as described [23]. Lumbar vertebrae were decalcified in ethylene-diamine tetraacetic acid (EDTA) glycerol solution for 5–7 days at 4°C. Decalcified samples were dehydrated and embedded in paraffin after which 5 µm sections were cut on a rotary microtome. The sections were stained with hematoxylin and eosin (H&E) or histochemically for total collagen, ALP and tartrate-resistant acid phosphatase (TRAP) as described below. Undecalcified bones were embedded in LR White acrylic resin (London Resin Co. Ltd., Theale, UK). Sections of 1 µm were cut on an ultramicrotome and stained for mineral with the von Kossa staining procedure and using toluidine blue as counter stain.

### Histochemical staining for collagen, ALP and TRAP

Total collagen was detected in paraffin sections using a modified method of Lopez-De Leon and Rojkind [24]. Dewaxed sections were exposed to 1% sirius red in saturated picric acid for 1h. After washing with distilled water, the sections were counterstained with hematoxylin and mounted with Biomount medium.

Enzyme histochemistry for ALP activity was performed as described [25]. Briefly, following preincubation overnight in 100 mM MgCl_2_ in 100 mM Tris-maleate buffer (pH 9.2), de-waxed sections were incubated for 2 hours at room temperature in 100 mM Tris-maleate buffer containing naphthol AS-MX phosphate (0.2 mg/ml, Sigma) dissolved in ethylene glycol monomethyl ether (Sigma) as substrate and fast red TR (0.4 mg/ml, Sigma) as a stain for the reaction product. After washing with distilled water, the sections were counterstained with Vector methyl green nuclear counterstain (Vector laboratories) and mounted with Kaiser's glycerol jelly.

Enzyme histochemistry for TRAP was performed using a modification of a previously described protocol [26]. Dewaxed sections were preincubated for 20 min in buffer containing 50 mM sodium acetate and 40 mM sodium tartrate at pH 5.0. Sections were then incubated for 15 min at room temperature in the same buffer containing 2.5 mg/ml naphthol AS-MX phosphate (Sigma) in dimethylformamide as substrate and 0.5 mg/ml fast garnet GBC (Sigma) as a color indicator for the reaction product. After washing with distilled water, the sections were counterstained with methyl green and mounted in Kaiser's glycerol jelly.

### Double calcein labeling

Double calcein labeling was performed by intraperitoneal injection of mice with 10 µg calcein/g body weight (C-0875, Sigma-Aldrich Corp., St. Louis, MO) at 10 and 3 day before death. Bones were harvested and embedded in LR White acrylic resin. Serial sections were cut, and the freshly cut surface of each section was viewed and imaged using fluorescence microscopy. The double calcein-labeled width of bone was measured using Northern Eclipse image analysis software version 6.0 (Empix Imaging, Inc., Mississauga, Canada), and the mineral apposition rate (MAR) was calculated as the interlabel width/labeling period.

### RNA isolation and Quantitative real-time PCR

RNA was isolated from 14-day cultured cells using Trizol reagent (Invitrogen) according to the manufacturer's protocol. Femurs were isolated and weighted. Homogenize tissue samples in 1 ml of TRIzol reagent per 50–100 mg of tissue using mortar and pestle with liquid nitrogen. After homogenization, remove insoluble material from the homogenate by centrifuging at 12,000 RPM for 10 min at 4°C. Transfer the top layer solution into a new tube. RNA of this solution was precipitated with isopropanol, washed with 75% ethanol and dissolved with DEPC water [27]. Reverse transcription reactions were performed using the SuperScript First-Strand Synthesis System (Invitrogen) as previously described [22]. To determine the number of cDNA molecules in the reverse transcribed samples, real-time PCR analyses were performed using the LightCycler system (Roche, Indianapolis, IN) as previously described [19].

### Western blot analysis

Purified hPTHrP1-34 and 1-84 and proteins extracted from kidney were quantitated by a kit (Bio-Rad, Mississauga, Ontario, Canada). Thirty µg protein samples were fractionated by SDS-PAGE and transferred to nitrocellulose membranes. Immunoblotting was carried out as described [22] using antibodies against PTHrP1-34, TRPV5 (ECaC1), calbindin-D_9K_ and calbindin-D_28K_, Na^+^/Ca^2+^ exchanger 1 (NCX1), and β-tubulin (Santa Cruz, CA, USA). Bands were visualized using ECL chemiluminescence (Amersham) and quantitated by Scion Image Beta 4.02 (Scion Corporation, NIH).

### Computer-assisted image analysis

After HE staining or histochemical staining of sections from six mice of each genotype, images of fields were photographed with a Sony digital camera. All images were digitally recorded using a rectangular template, and recordings were processed and analyzed using Northern Eclipse image analysis software as described [23], [26], [28].

### Statistical analysis

Data from image analysis are presented as means ± SEM. Statistical comparisons were made using Student's t-test, with P<0.05 being considered significant.

## Results

### Construction and expression of *hPTHrP1-34* and *1-84* in *pGEX-2TK*

**Figure 1:**
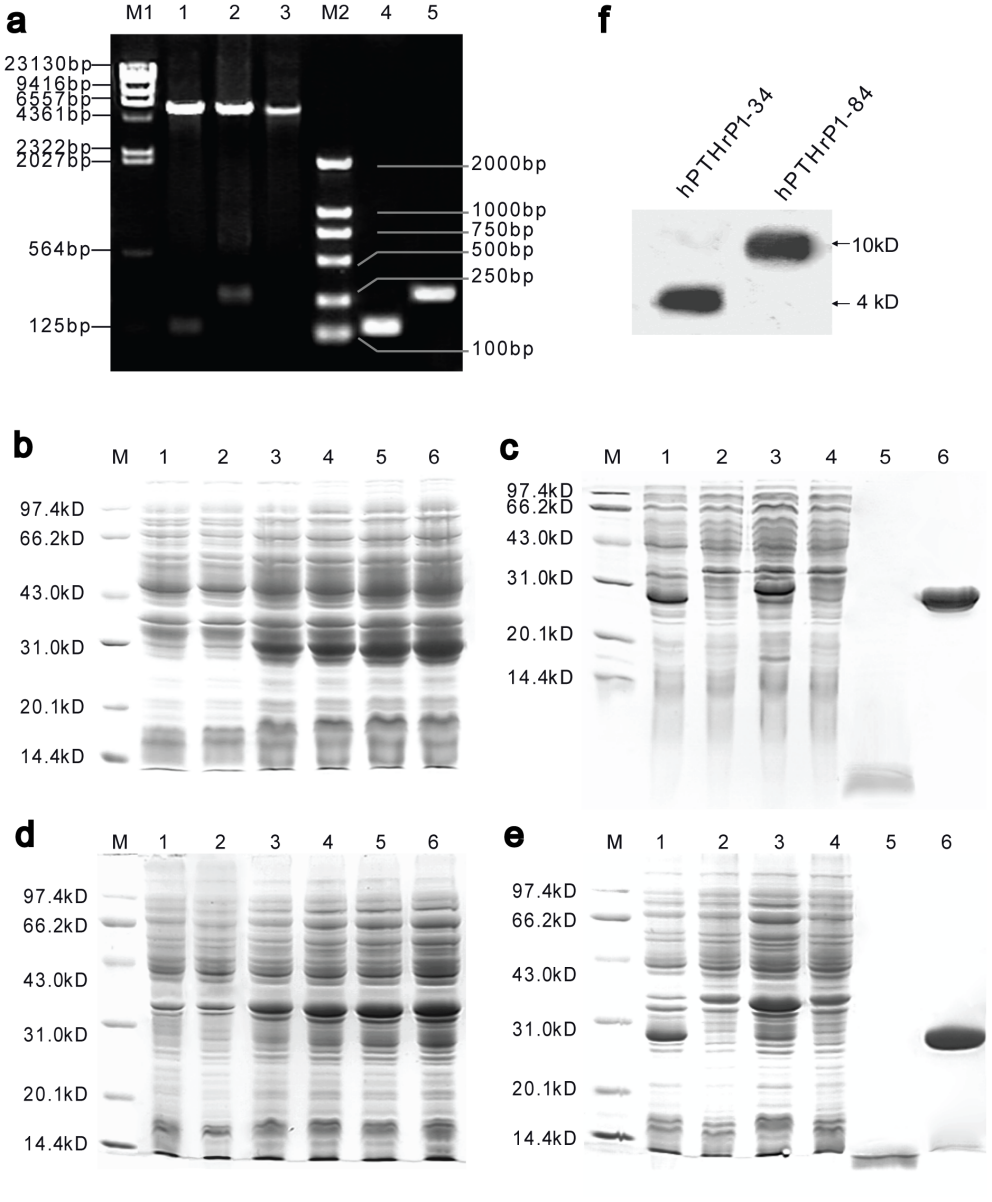
Preparation of Recombinant hPTHrP1-34 and 1-84. (a) Determination of the recombinants by PCR and double-enzyme (EcoR I+ BamH I) digestion. LaneM1, l-Hind III digest DNA marker; Lane1-3, double-enzyme digestion patterns of recombinant *hPTHrP1-34*, *hPTHrP1-84* and *pGEX-2TK*; LaneM2, DL2000 DNA marker; Lane4-5, PCR band patterns of recombinant hPTHrP1-34 and hPTHrP1-84. SDS-PAGE analysis of the GST-hPTHrP1-34 (b) or GST-hPTHrP1-84 (d) fusion protein under inducing condition and stained with coomassie blue. LaneM, molecular weight marker; Lane1, total cell soluble protein of BL21, control; Lanes2–6, cells with *pGEX-2TK/hPTHrP1-34* or *pGEX-2TK/hPTHrP1-84* after inducing with IPTG 0 h, 1 h, 2 h, 3 h, 4 h respectively. SDS-PAGE analyses of hPTHrP1-34 (c) or hPTHrP1-84 (e) peptide expression and purification. LaneM, molecular weight marker; Lane1, IPTG induction of BL21 transformed with *pGEX-2TK* without insert (expression control); Lane2, BL21 transformed with *pGEX-2TK/hPTHrP1-34* or *pGEX-2TK/hPTHrP1-84* without IPTG induction (induction control); Lane3, IPTG induction of BL21 transformed with *pGEX-2TK/hPTHrP1-34* or *pGEX-2TK/hPTHrP1-84*; Lane4, The proteins not bound to GSTrap FF column; Lane5, Purified hPTHrP1-34 or hPTHrP1-84 obtained after binding to GSTrap FF column and cleaved by thrombin; Lane6, GST-tag eluted from GSTrap FF column. (f) Western blot analysis of purified recombinant hPTHrP1-34 and hPTHrP1-84 with N-terminal hPTHrP antibody.

The recombinant expression vectors *pGEX-2TK/hPTHrP1-34* were used to transform competent E. coli BL21 (DE3) and five randomly picked ampicillin resistant colonies were grown. Plasmids were extracted and identified by PCR, and confirmed by double restriction enzyme digestion and sequencing (Fig. 1a). All of the selected transformants expressed an IPTG inducible protein which located in the expected site on SDS-PAGE. The optimal concentration of IPTG determined for protein induction is 1 mM (results not shown). Among the selected colonies, the one exhibited highest level of soluble expression was chosen for subsequent optimization studies and scale-up. Construction of *hPTHrP1-84* in *pGEX-2TK* followed the same procedures as described above.

The GST-hPTHrP1-34 and GST-hPTHrP1-84 fusion protein were both expressed mostly as soluble forms. Upon induction with IPTG, the fusion protein expression levels were continuously increased until reached a maximum concentration 3 hours later. The expression levels were increased with time of cultures after the induction (Figs. 1b and d). Therefore, we harvested the culturing engineered bacteria at 3 hours after the induction for more soluble fusion protein production.

### Purification and characterisation of recombinant hPTHrP1-34 and 1-84

Scale up production was carried out in 1 liter medium using the optimal conditions identified by small scale production (3-hour-induction with 1 mM IPTG). The 30-kDa GST-hPTHrP1-34 and 36-kDa GST-hPTHrP1-84 fusion protein were bound to Glutathione Sepharose 4B columns as described in Materials and Methods. After thrombin cleavage, 4-kDa hPTHrP1-34 and 10-kDa hPTHrP1-84 were released from the matrix (Figs. 1c and e). The total amount of purified soluble hPTHrP1-34 and 1-84 productions were varied from 100 to 200 µg/liter of cultures. Western blotting of the purified protein using N-terminal hPTHrP antibody revealed that purified hPTHrP1-34 and 1-84 both had immunologic activities (Fig. 1f).

### Recombinant hPTHrP1-34 and 1-84 promotes osteoprogenitor commitment to osteogenic lineage

**Figure 2:**
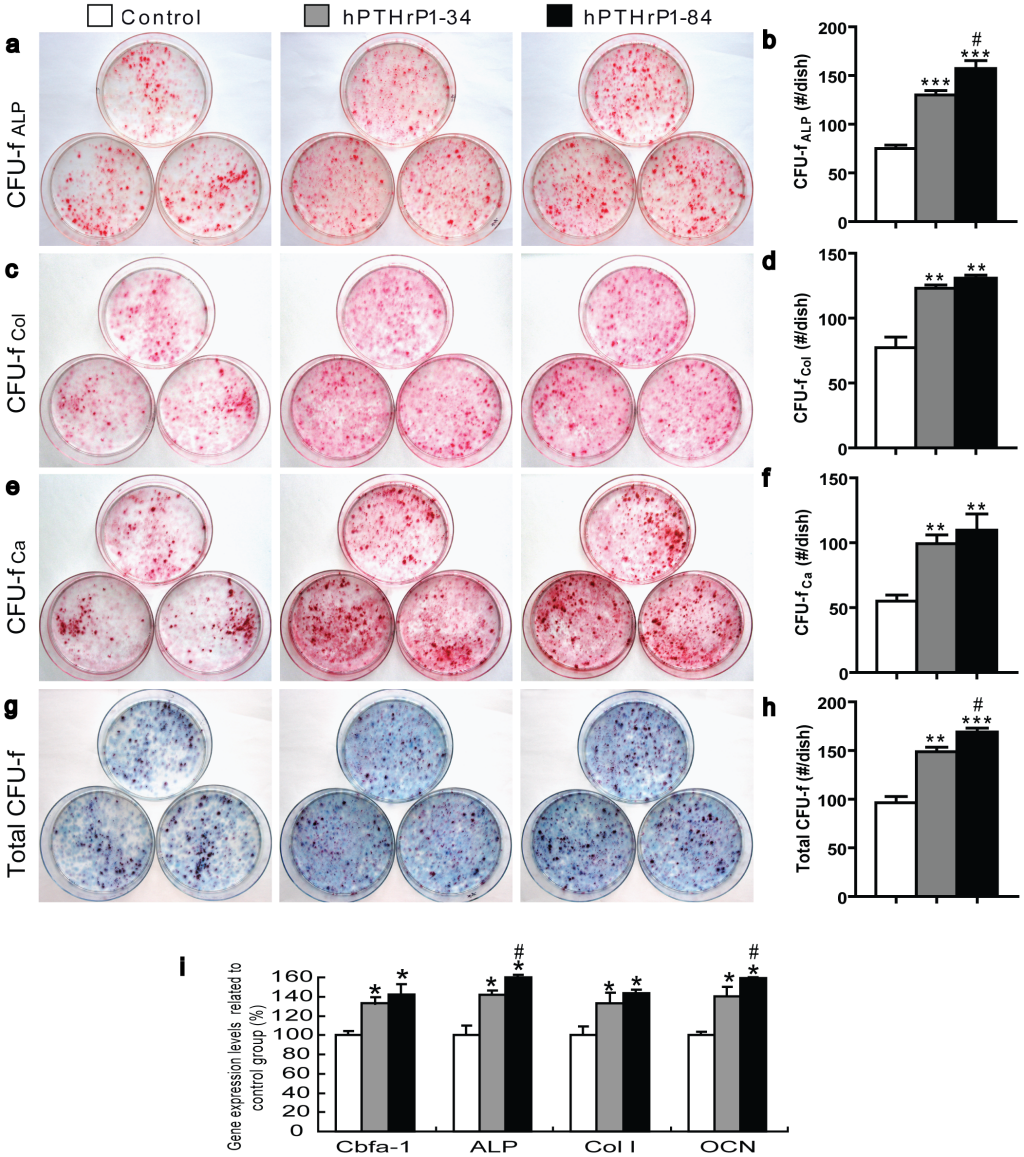
Effects of recombinant hPTHrP1-34 and 1-84 on BMCs. Cells from 18-day primary BMC cultures incubated in the absence (Control) or presence of 10^−7^ M hPTHrP1-34 (hPTHrP1-34) or presence of 10^−7^ M hPTHrP1-84 (hPTHrP1-84) on day 0 were stained cytochemically for ALP (a. CFU-f _ALP_) and with sirius red for total collagen(c. CFU-f _Col_) and with the von Kossa method for calcified colonies(e. CFU-f _Ca_) and with methylene blue to show total CFU-f (g. Total CFU-f) as described in Materials and Methods. The positive number of CFU-f _ALP_ (b), CFU-f _Col_ (d), CFU-f _Ca_ (f) and Total CFU-f number (h) per dish are the mean±SEM of triplicate determinations from three replicate experiments, respectively. (i) Real-time RT-PCR of cells extracts from 14-day primary BMC cultures for the expression of Cbfa I, ALP, Col I and OCN. Messenger RNA expression assessed by real-time RT-PCR is calculated as a ratio to the GAPDH mRNA level and expressed relative to levels of Control group. Each value is the mean ± SEM of three trials. *, P<0.05; **, P<0.01; ***, P<0.001 compared with Control group; #, P<0.05 compared with hPTHrP1-34 group.

To determine actions of recombinant hPTHrP1-34 and 1-84 on osteoprogenitor commitment, the primary bone marrow cultures were performed. The cells resulting cultures were examined for colony formation, and for markers of osteogenic differentiation including ALP activity, total collagen content, and mineralization of extracellular matrix. The results showed that treatment with hPTHrP1-34 and 1-84 at day 0 caused a significant increase in colony formation and osteogenic cell differentiation and mineralization compared with control cultures (Figs. 2a-h). Whereas it is noteworthy that the number of total colony forming units fibroblastic (CFU-f) and ALP-positive CFU-f were increased in hPTHrP1-84 treated cultures compared to hPTHrP1-34 treated cultures (Figs. 2a–h). We also examined the alterations of expression levels of genes related to osteogenic differentiation and mineralization. RNA was isolated from 14-day primary bone marrow cells (BMC) cultures and real time RT-PCR was performed. Results showed that gene expression levels of *Cbfa-1*, *ALP*, *type I collagen (Col I)* and *osteocalcin (OCN)* were upregulated significantly in hPTHrP1-34 and 1-84 treated primary BMC cultures compared with control cultures (Fig. 2i). These alterations were consistent with the osteogenic differentiation and mineralization observed by morphocytological analyses.

### Effects of recombinant hPTHrP1-34 and 1-84 on serum chemistry and on expression levels of calcium transporters in kidney

**Figure 3:**
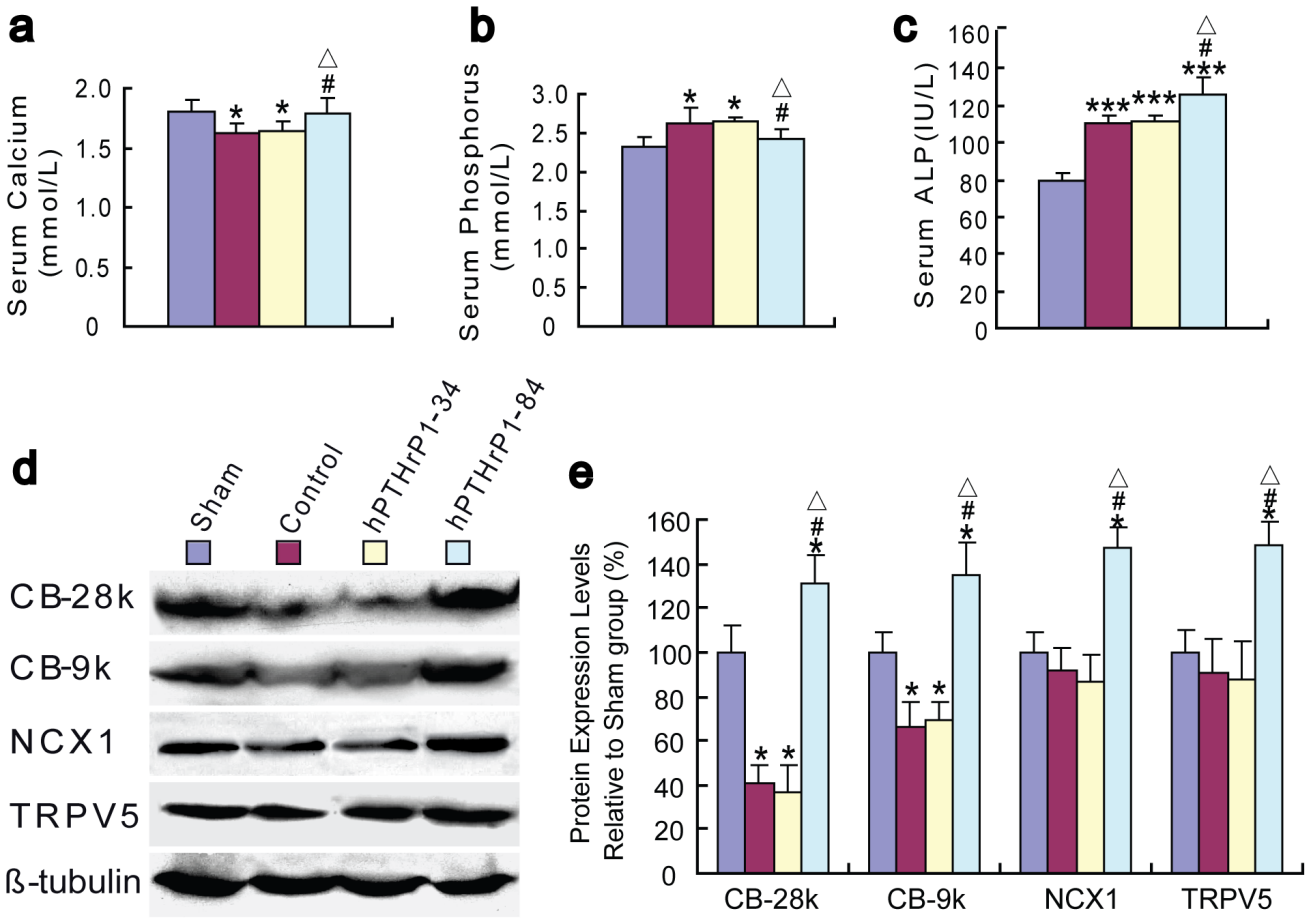
Effects of recombinant hPTHrP1-34 and 1-84 on serum chemistry and on expression of calcium transporters in kidney. (a) Serum calcium, (b) phosphorus and (c) ALP ratio were determined after 4-week administration in vehicle-treated sham-operated mice (Sham), vehicle-treated OVX mice (Control), hPTHrP1-34-treated OVX mice (hPTHrP1-34) and hPTHrP1-84-treated OVX mice (hPTHrP1-84). (d) Western blots of renal extracts for expression of CB_28K_, CB_9K_, NCX1 and TRPV5. β-tubulin was used as loading control for Western blots. (e) CB_28K_, CB_9K_, NCX1 and TRPV5 protein levels relative to β-tubulin protein level and expressed relative to levels of Sham group. Each value is the mean ± SEM of determinations in six mice of each group.*, P<0.05; ***, P<0.001 compared with Sham group; #, P<0.05 compared with Control group; △, p<0.05 compared with hPTHrP1-34 group.

To determine whether the administrations of recombinant hPTHrP1-34 and 1-84 altered serum mineral ion levels and osteoblast activity in OVX mice, serum calcium (Fig. 3a) and phosphorus (Fig. 3b) and bone specific ALP (Fig. 3c) were examined at 4 weeks after the drug administration. Serum ALP levels, most likely reflect osteoblast stimulation. The vehicle-treated OVX mice displayed hypocalcemia, hyperphosphatemia, and increased serum ALP levels compared with vehicle-treated sham-operated mice. Serum mineral ion and ALP levels had no significant differences between vehicle-treated and hPTHrP1-34-treated OVX mice. Whereas recombinant hPTHrP1-84-treated OVX mice displayed normocalcemia, normophosphatemia, and more obviously ALP elevations compared with vehicle-treated sham-operated mice (Figs. 3a–c).

To determine whether the raised serum calcium levels induced by recombinant hPTHrP1-84 were due to increased renal reabsorption of calcium, we examined the protein expression levels of the renal calcium transporters by Western blot (Fig. 3d). The results revealed that the protein levels of calbindin-D_28K_, calbindin-D_9K_, NCX1, and TRPV5 were down-regulated in the kidney of vehicle-treated and hPTHrP1-34-treated OVX mice relative to vehicle-treated sham-operated mice,however, they were up-regulated dramatically by hPTHrP1-84 treatment (Figs. 3d and 3e).

### Effects of recombinant hPTHrP1-34 and 1-84 on bone volume

**Figure 4:**
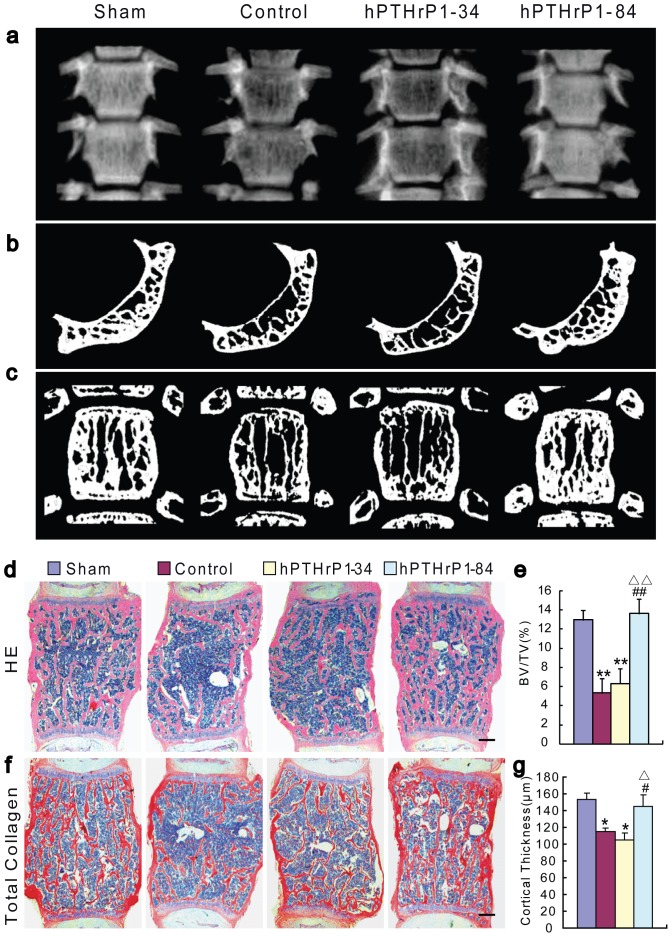
Effects of recombinant hPTHrP1-34 and 1-84 on bone volume. (a) Representative contact radiographs of thoracic vertebrae. Representative (b) cross-sections and (c) longitudinal sections of 3D reconstructed thoracic vertebrae. Micrographs of decalcified paraffin sections of lumbar vertebrae stained with H&E (d) and total collagen (f). Scale bars represent 200 µm in d and f. Trabecular bone volume relative to the tissue volume [BV/TV (%), e] and the cortical thickness (g) of vertebrae were determined by histomorphometric analysis as described in Materials and Methods. Each value is the mean ± SEM of determinations in six mice of each group. *, P<0.05; **, P<0.01 compared with Sham group; #, P<0.05; ##, P<0.01 compared with Control group; △, P<0.05; △△, P<0.01 compared with hPTHrP1-34 group.

To assess whether the administrations of recombinant hPTHrP1-34 and 1-84 could prevent estrogen deficiency induced bone loss, bone mineral density and bone volume were analyzed in vertebrae by radiography (Fig. 4a), micro-CT (Figs. 4b and 4c), histology (Fig. 4d) and histochemistry for total collagen (Fig. 4f). The results revealed that the radiolucency was increased in vertebrae in vehicle-treated and hPTHrP1-34-treated OVX mice, was normalized in hPTHrP1-84-treated OVX mice compared to vehicle-treated sham-operated mice (Fig. 4a). This was further verified by 3D micro-CT scanning and histological analysis, which demonstrated that trabecular bone volume and cortical thickness were decreased dramatically in vehicle-treated OVX mice compared to vehicle-treated sham-operated mice. Both parameters were not altered significantly in hPTHrP1-34-treated OVX mice, but were increased significantly in hPTHrP1-84-treated OVX mice compared to vehicle-treated and hPTHrP1-34-treated OVX mice (Figs. 4b–g). We also examined the alterations of bone volume in tibiae, which were consistent with the results observed in vertebrae (Figs. S1a and S1c).

### Effects of recombinant hPTHrP1-34 and 1-84 on osteoblastic bone formation

**Figure 5:**
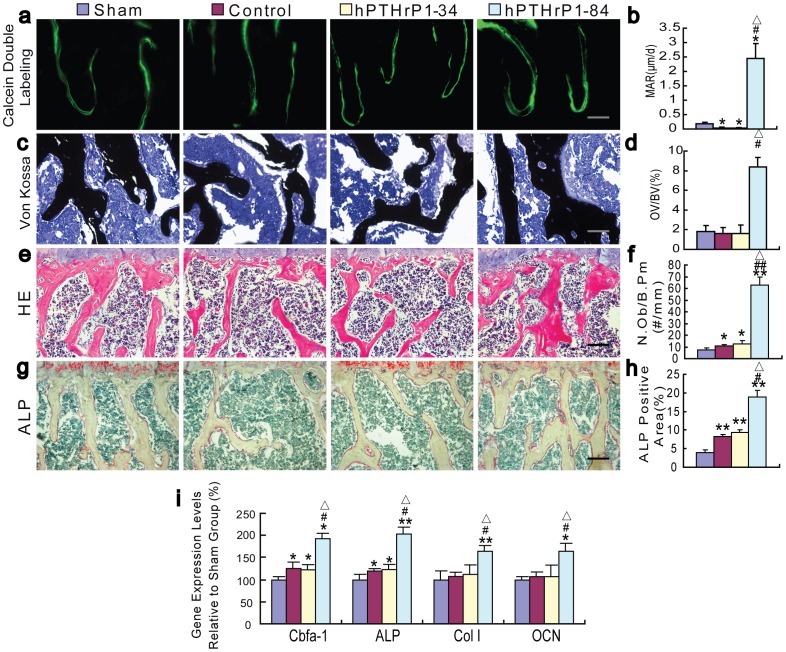
Effects of recombinant hPTHrP1-34 and 1-84 on osteoblastic bone formation parameters. (a) Representative micrographs of calcein double labeling and (c) sections stained with the von Kossa procedure in the trabeculae were imaged from ethanol fixed and undecalcified LR white resin embedded sections of vertebrae. (b) MAR of trabeculae was determined. (d) Osteoid volume was determined in undecalcified von Kossa-stained sections and is presented as a percent of bone volume (OV/BV, %) of trabeculae. Micrographs of decalcified paraffin sections of vertebrae stained with H&E (e) and histochemically for ALP (g). (f) Number of osteoblasts per mm bone parameter (N.Ob/B.Pm, #/mm) and (h) ALP positive area as a percent of the tissue area were determined in the vertebrae. Scale bars represent 25 µm in a and c and 50 µm in e and g. (i) Real-time RT-PCR of long bone extracts for the expression of *Cbfa I*, *ALP*, *Col I* and *OCN*. Messenger RNA expression assessed by real-time RT-PCR is calculated as a ratio to the *GAPDH* mRNA level and expressed relative to levels of Sham group. Each value is the mean ± SEM of determinations in six mice of each group. *, P<0.05; **, P<0.01 compared with Sham group; #, P<0.05; ##, P<0.01 compared with Control group; △, P<0.05 compared with hPTHrP1-34 group.

To determine whether the alterations of bone volume were associated with those of osteoblastic bone formation, MAR (Fig. 5a), histomorphometric analysis for osteoid volume (Fig. 5c), osteoblast number (Fig. 5e), and ALP activity (Fig. 5g) were examined in vertebrae at 4 weeks after the drug administration. Results showed that the MAR was decreased, osteoid volume was not altered significantly, whereas osteoblast number and ALP positive area were increased in vehicle-treated and hPTHrP1-34-treated OVX mice compared to vehicle-treated sham-operated mice (Figs. 5a–h). All parameters for osteoblastic bone formation including MAR, osteoid volume, osteoblast number and ALP positive area (Figs. 5a–h) were increased dramatically in hPTHrP1-84-treated OVX mice compared with the other groups. Consistent with these observations, osteoblastic gene expression levels of *Cbfa1*, *ALP* were slightly up-regulated in vehicle-treated and hPTHrP1-34-treated OVX mice compared to vehicle-treated sham-operated mice; however, *Cbfa1*, *ALP*, *Col I*, and *OCN* were all up-regulated significantly in hPTHrP1-84-treated OVX mice compared to the other groups as demonstrated by real-time RT-PCR (Fig. 5i). We also examined the alterations of osteoblast number in tibiae, which were consistent with the results observed in vertebrae (Figs. S1b and S1d).

### Effects of recombinant hPTHrP1-34 and 1-84 on osteoclastic bone resorption

**Figure 6:**
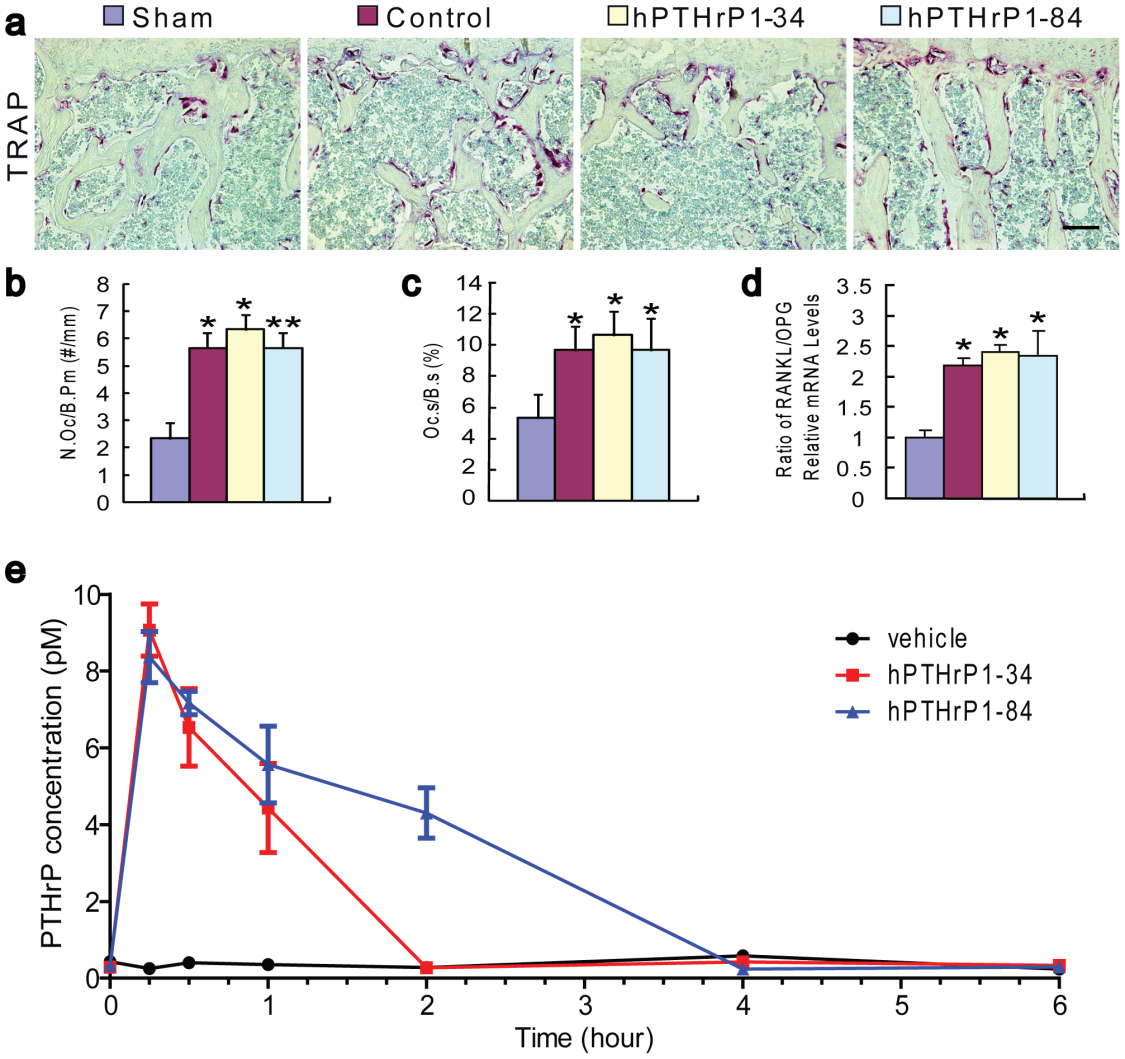
Effects of recombinant hPTHrP1-34 and 1-84 on osteoclastic bone resorption parameters and half-life analyses of recombinant hPTHrP1-34 and 1-84 in vivo. (a) Representative micrographs of paraffin embedded sections of vertebrae stained histochemically for TRAP and photographed at a magnification of 200. Scale bars in a represents 50 µm. (b) Number of TRAP positive osteoclasts per mm bone parameter (N.Oc/B.Pm, #/mm) and (c) the surface of osteoclasts relative to the bone surface (Oc.S/B.S, %) were determined in the trabeculae of TRAP-stained vertebrae. Each value is the mean ± SEM of determinations in 6 animals of each group. (d) Real-time RT-PCR was performed on long bone extracts for *RANKL* and *OPG* mRNA as described in Materials and Methods. Messenger RNA expression assessed by real-time RT-PCR analysis is calculated as a ratio to the *GAPDH* mRNA level and expressed relative to levels of Sham group. Ratio of *RANKL/OPG* relative mRNA levels was calculated and was presented as the mean ± SEM of determinations in six animals of each group. (e) Plasma immunoreactive PTHrP determined using ELISA after subcutaneous injection of vehicle or hPTHrP1-34 or hPTHrP1-84. Blood samples were drawn at the following time points after injection of study drugs: 15 and 30 minutes, then 1, 2, 4, 6 hours. Each value is the mean ± SEM of determinations in 6 animals of each group. *, P<0.05; **, P<0.01 compared with Sham group.

To determine whether hPTHrP1-34 and 1-84 exerted catabolic effects on bone in OVX mice, histochemical staining for TRAP (Fig. 6a) were performed and osteoclast number and surface (Figs. 6b and 6c) were determined by image analysis. The results revealed that TRAP positive osteoclast number and surface were increased significantly in vehicle-treated OVX mice compared to vehicle-treated sham-operated mice. Whereas hPTHrP1-34 and 1-84 administration did not further increase TRAP positive osteoclast number and surface in OVX mice compared with vehicle-treated OVX mice (Figs. 6a–c). The ratios of *RANKL/OPG* mRNA levels were increased significantly in vehicle-treated, hPTHrP1-34-treated and hPTHrP 1-84-treated OVX mice compared to vehicle-treated sham-operated mice, however, they had no significant differences among vehicle-treated, hPTHrP1-34-treated and hPTHrP 1-84-treated OVX mice as demonstrated by real-time RT-PCR (Fig. 6d).

### Half-life of recombinant hPTHrP1-34 and 1-84 in vivo

To assess whether the different effects of recombinant hPTHrP1-34 and 1-84 in vivo were associated with the differences of their half-life (t_1/2_) in vivo, serum concentrations of hPTHrP1-34 and hPTHrP1-84 were measured at different time points after their administration. Results showed that plasma PTHrP levels were lower than 0.5 pM at all observed time points in vehicle-treated OVX mice, and rose to 9±0.7 and 8.5±0.7 pM at 15 min after the administration in hPTHrP1-34 and hPTHrP1-84-treated OVX mice, respectively. Then plasma PTHrP levels declined gradually until baseline levels at 2 hours in hPTHrP1-34-treated OVX mice, and 4 hours in hPTHrP1-84-treated OVX mice (Fig. 6e). From the figure, it can be seen that the circulating t_1/2_ of hPTHrP1-34 was approximately 45 mins, whereas the t_1/2_ of hPTHrP1-84 was approximately 2 hours.

## Discussion

Recombinant hPTHrP1-34 and 1-84 were expressed using the GST Gene Fusion System, allowing purification of free recombinant peptide in a single affinity chromatographic step. The results of DNA sequence analysis showed that the gene sequences constructed for the recombinant hPTHrP1-34 and 1-84 were correct templates for expressing them. The molecular weights obtained by SDS-PAGE were the same as the theoretical value calculated from the atomic weight sum of atoms which consists the peptide according to the amino acid sequence. The results of the Western blotting revealed that the recombinant hPTHrP1-34 and 1-84 had immunological activities. Then, we employed them to investigate their actions in promoting BMC commitment to osteogenic lineage and stimulating bone formation.

PTHrP is a ubiquitously produced local paracrine, autocrine and intracrine factor, the role of which is to regulate cellular differentiation, proliferation, cell death, and epithelial calcium transport, both during development as well as in adult life [29]–[30]. Osteoblasts are also target cells for locally produced PTHrP and circulating PTH, as these cells express PTHR1 [31]–[33]. Our previous results demonstrated that PTHrP stimulates osteogenic cell proliferation in rat marrow mesenchymal progenitor cells through protein kinase C-dependent activation of the Ras and MAPK signaling pathway [34]. Several groups have demonstrated that PTHrP can influence osteoblast proliferation, differentiation and function [35]. Consistent with these results, our data also showed that treatment with hPTHrP1-34 and 1-84 caused a significant increase in colony formation and osteogenic cell differentiation and mineralization in primary bone marrow cultures compared with control cultures. We also found that treatment with PTHrP1-84 resulted in a slightly increase in total CFU-f number and ALP-positive CFU-f number compared with PTHrP1-34 treated cultures. Our findings indicate that both of recombinant hPTHrP1-34 and 1-84 enhance BMC commitment to osteogenic lineage, and the effect of recombinant hPTHrP1-84 is a little stronger than hPTHrP1-34. We have mentioned that Mid-region of PTHrP interact with a yet unidentified receptor to maintain matemo-foetal gradients of calcium [29], [36]. So we presume that the Mid-region of PTHrP probably exerts a major influence on calcium transport in hPTHrP1-84-treated primary bone marrow cultures. However, exact mechanism remains to be determined.

In previous work, we have shown that 3-month-old mice heterozygous for the *Pthrp*-null allele exhibit a form of skeletal haploinsufficiency characterized by decreased bone volume and bony structural alterations consistent with premature, advanced osteoporosis [37]. Our previous studies in mice with osteoblast-specific targeted disruption of PTHrP showed that osteoblast-derived PTHrP is a potent endogenous bone anabolic factor which potentiates bone formation by altering osteoblast recruitment and survival [37]. All these evidences support that PTHrP is a potent endogenous bone anabolic agent. With regard to the action of exogenous PTHrP1-86 on skeletal alteration, we have shown that PTHrP increased cortical and trabecular bone mass with augmented osteoblast number and activity; however, bone resorption was not increased [19]. Consistent with these results, our data also demonstrated that recombinant hPTHrP1-84 administration increased bone mass in osteoporosis mice by stimulating osteoblastic bone formation. After subcutaneously injected hPTHrP1-84 into osteoporosis animal models for 4 weeks, all osteoblastic bone formation parameters including MAR, osteoid volume, osteoblast number, ALP positive area and osteoblastic gene expression levels were increased dramatically in hPTHrP1-84-treated OVX mice compared with the vehicle-treated OVX mice. These results suggest that recombinant hPTHrP1-84 is an effective agent to stimulate bone formation.

Unexpectedly, unlike recombinant hPTHrP1-84, the recombinant hPTHrP1-34 administration did not increased bone mass and osteoblastic bone formation parameters in OVX mice in vivo. We also found that serum calcium and renal calcium transporters were not alterd by hPTHrP1-34 administration, but were increased significantly by hPTHrP1-84 administration in OVX mice. The transcellular calcium transport involves many molecules, including TRPV5 as the apical entry gate for Ca^2+^, calbindin-D_9K_ and calbindin-D_28K_ as intracellular ferry proteins for Ca^2+^, and NCX1 as Ca^2+^ extrusion systems across the basolateral membrane to the blood compartment [38]. Our results revealed that the protein levels of renal calcium transporters, including calbindin-D_9K_, calbindin-D_28K_, NCX1, and TRPV5 were all down-regulated in the kidney of vehicle-treated and hPTHrP1-34-treated OVX mice compared with vehicle-treated sham-operated mice, however their levels were up-regulated significantly by hPTHrP1-84 administration. These data suggest that raised serum calcium levels induced by recombinant hPTHrP1-84 were due to increased renal reabsorption of calcium. In previous studies, 1,25(OH)_2_D_3_ and the vitamin D receptor have been reported to modulate calcium transporters in vivo [39]-[40]. Our previous study has demonstrated that, similar to PTH1-34, PTHrP1-86 administration up-regulated renal calcium transporters in vivo [19]. Our results indicate that hPTHrP1-84 could modulate renal calcium transporters. In view of the fact that bone resorption was not altered by hPTHrP1-84 administraion, the major mechanism of raised serum calcium levels were mediated by stimulating renal calcium reabsorption. Our previous studies suggest that increased extracellular calcium by endogenous PTH [41] or 1,25(OH)_2_D_3_ [42] can increase bone mass and stimulate osteoblastic bone formation mediated by calcium sensing receptor (CaSR). In tissue culture models, elevations in extracellular free ionized calcium concentrations increase osteoblast chemotaxis and proliferation [43]-[44], and alter the levels of expression of some differentiation markers [45]. Furthermore, deletion of both alleles of the Casr gene in osteoblasts profoundly blocked postnatal growth and skeletal development, a finding that was evident by 3 days of age [46]. These results support that hPTHrP1-84, at least partly, can stimulate osteoblastic bone formation and increase bone mass by increasing extracellular calcium levels mediated by CaSR.

In order to explore possible reason why recombinant hPTHrP1-34 and 1-84 displayed different response in vivo, we examined whether their different effects were associated with the differences of their half-life in vivo. By measured serum concentrations of hPTHrP1-34 and hPTHrP1-84 at different time points after their administration, we found that the circulating lifetime of hPTHrP1-34 is much shorter than hPTHrP1-84, suggesting that hPTHrP1-34 may be degraded more rapidly than hPTHrP1-84 in vivo. On the other hand, it is also possible that hPTHrP1-34 has less affinity with PTHR, which remains to be investigated.

In summary, in this study, GST Gene Fusion System was used to express recombinant hPTHrP1-34 and 1-84. *HPTHrP1-34* and *1-84* gene fragments were cloned into the *pGEX-2TK* vector, the host cells used for the cloning steps were transformed, and the presences of the insert were verified. After scale up production, recombinant hPTHrP1-34 and 1-84 were obtained by means of cell disruption, Glutathione Sepharose 4B column purification and thrombin digestion. Examined activities of recombinants in vitro, we found that treatment of hPTHrP1-34 and 1-84 caused a significant increase in colony formation and osteogenic cell differentiation and mineralization in primary bone marrow cultures; however, the effect of recombinant hPTHrP1-84 is a little stronger than hPTHrP1-34. Next, ovariectomy was used to construct osteoporosis animal model to test activities of recombinants in vivo. HPTHrP1-84 administration elevated extracellular calcium levels by increasing renal calcium transport, which resulted in stimulation of osteoblastic bone formation. These factors contributed to augmented bone mass in hPTHrP1-84-treated OVX mice but did not affect bone resorption. There was no obviously bone volume alteration in hPTHrP1-34 treated OVX mice. For this explanation, we found that the circulating lifetime of hPTHrP1-34 is much shorter than hPTHrP1-84, suggesting that hPTHrP1-34 may be degraded more rapidly than hPTHrP1-84 in vivo. This study implies that recombinant hPTHrP1-84 is more effective than hPTHrP1-34 to enhance renal calcium reabsorption and to stimulate osteoblastic bone formation.

## Supporting Information

Figure S1 **Effects of recombinant hPTHrP1-34 and 1-84 on the phenotype of tibiae.** Micrographs of decalcified paraffin sections of tibiae stained with total collagen (a) and H&E (b). (c) Trabecular bone volume relative to the tissue volume [BV/TV (%)] and (d) number of osteoblasts per mm bone parameter (N.Ob/B.Pm, #/mm) were determined by histomorphometric analysis as described in Materials and Methods. Each value is the mean ± SEM of determinations in six mice of each group. *, P<0.05; **, P<0.01 compared with Sham group; #, P<0.05; ##, P<0.01 compared with Control group; △, P<0.05; △△, P<0.01 compared with hPTHrP1-34 group.
(TIF)
