# Supplementary figures and images for: Recombinant Human Parathyroid Hormone Related Protein 1-34 and 1-84 and Their Roles in Osteoporosis Treatment
Source: PLoS One. 2014 Feb 6;9(2):e88237. doi: 10.1371/journal.pone.0088237 (PMC3916416; doi:10.1371/journal.pone.0088237)

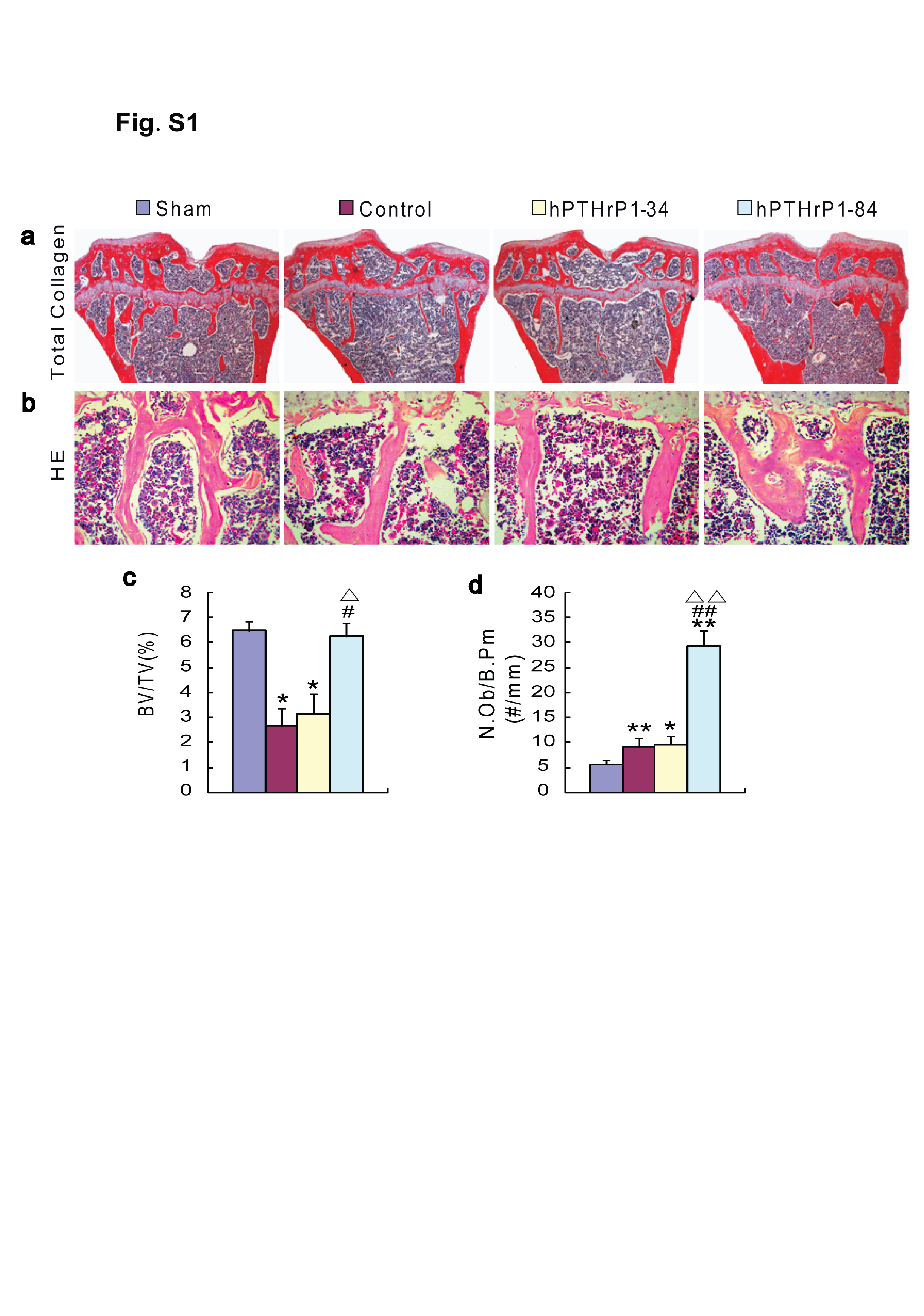

Supplement: Figure S1 — Effects of recombinant hPTHrP1-34 and 1-84 on the phenotype of tibiae. Micrographs of decalcified paraffin sections of tibiae stained with total collagen (a) and H&E (b). (c) Trabecular bone volume relative to the tissue volume [BV/TV (%)] and (d) number of osteoblasts per mm bone parameter (N.Ob/B.Pm, #/mm) were determined by histomorphometric analysis as described in Materials and Methods. Each value is the mean ± SEM of determinations in six mice of each group. *, P<0.05; **, P<0.01 compared with Sham group; #, P<0.05; ##, P<0.01 compared with Control group; △, P<0.05; △△, P<0.01 compared with hPTHrP1-34 group. (TIF) [file pone.0088237.s001.tif]
